# Impact of COVID-19 pandemic on science communication

**DOI:** 10.1590/2317-1782/20242024068en

**Published:** 2024-04-26

**Authors:** Ana Luiza Navas, Pamela Papile Lunardelo, Stela Maris Aguiar Lemos, Vanessa Veis Ribeiro, Leonardo Wanderley Lopes

**Affiliations:** 1 Faculdade de Ciências Médicas da Santa Casa de São Paulo - São Paulo (SP), Brasil.; 2 Programa de Pós-graduação em Psicobiologia - FFCL, Universidade de São Paulo - USP - Ribeirão Preto (SP), Brasil.; 3 Universidade Federal de Minas Gerais - UFMG - Belo Horizonte (MG), Brasil.; 4 Universidade de Brasília - UNB - Brasília (DF), Brasil.; 5 Universidade Federal da Paraíba - UFPB - João Pessoa (PB), Brasil.

The dissemination of scientific knowledge through digital media began at the end of the 1990s, years later, the publication of “Altmetrics: a manifesto” received special attention. The article published in 2010 presented the possibility of using the internet to measure the impact generated by scientific materials through their tracking on networks^(^1,2^)^. Until this time, these would be two major milestones in the phenomenon of dissemination of scientific knowledge via the internet.

The Editorial CoDAS “Divulgação científica como forma de compartilhar conhecimento”^(^3^)^ was published in June 2020, when the world entered a period of isolation due to COVID-19 pandemic. When the Editorial was submitted it was not yet possible to predict what would happen in the next 24 months, but without a doubt the use of social media has intensified and access to the internet has expanded^(^4^)^. Furthermore, the search for information based on scientific evidence also began to be carried out not only by researchers, but also by professionals and the general public. Communication and dissemination of knowledge has become more accessible and more valuable every day to guide decision-making.

Almost 4 years after the publication of Navas et al.^(^3^)^, we decided to describe the impact of the Covid-19 Pandemic on scientific dissemination, in the particular case of CoDAS journal, based on the comparison of CoDAS indicators pre and post pandemic.

The use of alternative metrics or altmetrics (ALTernative article-level metrics) continues to be considered as an estimate of the impact promoted by a given scientific product^(^5,6^)^. Based on articles published in Open Access, different data generating sources track the movement of scientific material in the digital environment, quantifying the number of online views, readings of the full text, number of downloads and mentions on blogs or social media^(^7^)^. The insights provided by social media allow us to understand the reach and impact of a scientific article in different dimensions in relation to traditional bibliometric metrics^(^2^)^.

The use of alternative metrics has advantages such as the speed of dissemination and feedback regarding the impact of the scientific article and the social elements promoted by the material (e.g. use in public policies, articles in newspapers for a broad audience). However, it is necessary to highlight that there are still challenges in the study of alternative metrics, but these do not preclude their use, such as the lack of standardization and consistency between the different sources that collect, aggregate and analyze data^(^8^)^. Although alternative metrics do not replace traditional metrics, they certainly add to them to provide new dimensions of the impact of a given scientific article on the scientific and social community^(^2^)^.

Given these assumptions, the CoDAS media communication team continues to disseminate articles published online and carries out monitoring on digital platforms, as an important indicator of the impact of scientific production for the specialized community and the general public.

CoDAS journal's activities on social media started in mid-2019 and by 2020, 69 publications referring to scientific articles published in this Journal had been made on social media. At this time, publications referring to scientific articles totaled 394 on the social networks Instagram, Twitter and Facebook and 36 publications on LinkedIn, the most recent social network, created in 2023.

On Instagram, between 2019 and 2020, CoDAS had 1,715 active followers with an average reach of 1,900 accounts and 2,600 impressions per publication. In 2023, the number of active followers increased to 5,060, the average publication reached 4,505 and the average number of impressions was 2,841, with a substantial increase in all indicators.

On the Twitter account, also created in 2019, there were 57 followers and an average number of 230 impressions per publication, in 2020. And on Facebook, the official CoDAS page had 1,231 active followers with an average reach per publication of 200 accounts, until 2020. In 2023, the number of active followers on Twitter was 118 and the average number of impressions per publication was 507. On Facebook the current number of followers is 2,200, with a decrease in the average publication reach to 147. It is necessary It should be noted that nationwide the use of Facebook, in general, has decreased in recent years, with its users migrating to Instagram.

The creation of an official LinkedIn account was based on the importance of this social network for work and academic relationships. Activities on LinkedIn began in July 2023 and so far 38 posts referring to scientific articles have been made, with an average reach of 1,193. There are 432 active followers, with an average of 60 profile views per week.

Instagram allows you to check the number of impressions (times the post was seen), the number of accounts reached, the engagement rate and demographic data. On this network, the CoDAS publication that promoted the largest number of impressions in 2022 was the article by Mouffron et al.^(^9^)^, entitled “Immediate effects of photobiomodulation on maximum lip pressure”. The publication that reached the most accounts was that of Nasciutti et al.^(^10^)^ with the article “Quality of life of the Brazilian speech therapist facing the covid-19 pandemic”. These same articles were those that received the highest number of likes in 2022, followed by “Dysphagia due to anterior cervical osteophytosis: case report” of Aires et al.^(^11^)^. In 2023, the publication that generated the highest number of impressions was “Therapeutic lying: Brazilian speech and language therapists’ point of view about a controversial communication strategy employed in the care for people with dementia” by Lopes et al.^(^12^)^. The article by Santos et al.^(^13^)^ entitled “Effects of lingual frenotomy on breastfeeding and electrical activity of the masseter and suprahyoid muscles” promoted the highest number of accounts reached. The highest number of likes occurred when the article was published “Effects of Kangaroo Care on the development of oral skills and achievement of exclusive oral feeding in preterm infants” by Ciochetto et al.^(^14^)^. On LinkedIn, the insights provided by the network are also based on the number of accounts reached and impressions. The publication that promoted the highest numbers was “Assessment protocol for acquired apraxia of speech” from Costa et al.^(^15^)^.

Twitter provides data on engagement per publication, and in 2022 the Letter to the Editor by Dornelas et al.^(^16^)^ entitled “Chronic Cough and Speech Therapy” promoted the largest engagement numbers. In the following year, the publication with the highest number of engagement was “Web version of the protocol of the orofacial myofunctional evaluation with scores: usability and learning.” of Ataide et al.^(^17^)^. On Facebook, the article with the highest number of publication engagement in 2022 was the same as on Instagram, while in 2023 the article by Ferrari et al.^(^18^)^ called “Risk of dysphonia and voice quality in performing arts students”.

Considering the different areas of speech therapy, the years 2022 and 2023 and all of the journal social networks, we highlight the publication with the highest number of shares in each specific area (Chart 1).

**Chart 1:** Publications with the highest number of shares by area of Speech Therapy

**Area of Specialty**	**Article Title**	**Authorship**
Voice	Reducing the GAP between science and clinic: lessons from academia and professional practice - part A: perceptual-auditory judgment of vocal quality, acoustic vocal signal analysis and voice self-assessment.	Behlau et al.^(^19^)^
Hearing and Balance	Frailty syndrome and risks for falling in the elderly community	Taguchi et al.^(^20^)^
Orofacial Motricity	Immediate effects of photobiomodulation on maximum lip pressure	Mouffron et al.^(^9^)^
Dysphagia	Dysphagia due to anterior cervical osteophytosis: case report	Aires et al.^(^11^)^
Speech	Translation into Brazilian Portuguese and transcultural adaptation of the Apraxia of Speech Rating Scale 3.5	Santos et al.^(^21^)^
Language	Efficacy in the use of gamification strategy in phonological therapy	Silva et al.^(^22^)^
Educational Speech Pathology	Cut-off point, sensitivity and specificity for screening the reading fluency in children	Cogo-Moreira et al.^(^23^)^
Public Health	Matricial support for community health agents on the auditory and language development milestones in early childhood	Silva and Silva^(^24^)^

The SBFa's mission is to promote, debate and disseminate scientific and professional production in the area, as well as discuss issues related to training in Speech Therapy and in this sense, CoDAS has been a valuable asset for the dissemination of scientific evidence for professional practice, for discussion of public policies and to strengthen speech therapy in Brazil.
