# Supplementary material for: Impact of COVID-19 pandemic on science communication
Source: Codas. 2024 Apr 26;36(2):e20240068. doi: 10.1590/2317-1782/20242024068en (PMC11086976; doi:10.1590/2317-1782/20242024068en)
Supplement: Translation

# Translation: O impacto da pandemia da COVID-19 para a divulgação científica

**DOI:** 10.1590/2317-1782/20242024068pt

**Authors:** Ana Luiza Navas, Pamela Papile Lunardelo, Stela Maris Aguiar Lemos, Vanessa Veis Ribeiro, Leonardo Wanderley Lopes

**Affiliations:** 1 Faculdade de Ciências Médicas da Santa Casa de São Paulo - São Paulo (SP), Brasil.; 2 Programa de Pós-graduação em Psicobiologia - FFCL, Universidade de São Paulo - USP - Ribeirão Preto (SP), Brasil.; 3 Universidade Federal de Minas Gerais - UFMG - Belo Horizonte (MG), Brasil.; 4 Universidade de Brasília - UNB - Brasília (DF), Brasil.; 5 Universidade Federal da Paraíba - UFPB - João Pessoa (PB), Brasil.

A divulgação do conhecimento científico pelos meios digitais iniciou-se no final da década de 1990, anos mais tarde, a publicação de “*Altmetrics: a manifesto*” recebeu especial atenção. O artigo publicado no ano de 2010 apresentava a possibilidade de uso da internet para mensurar o impacto gerado pelos materiais científicos por meio do seu rastreio nas redes^(^1,2^)^. Até esta ocasião, estes seriam dois grandes marcos do fenômeno de disseminação do conhecimento científico pela internet.

O Editorial CoDAS “Divulgação científica como forma de compartilhar conhecimento”^(^3^)^ foi publicado em junho de 2020, momento em que o mundo entrava em um período de isolamento por causa da pandemia de COVID-19. Na data de submissão do Editorial ainda não se podia prever o que aconteceria nos próximos quase 24 meses, mas sem dúvida o uso das mídias sociais se intensificou e o acesso à internet foi ampliado^(^4^)^. Além disso, a busca por informações baseadas em evidências científicas também passou a ser realizada não somente por pesquisadores, mas também por profissionais e pelo público geral. A comunicação e difusão do conhecimento, se tornou cada dia mais acessível e mais valiosa para guiar as tomadas de decisão.

Passados quase 4 anos da publicação de Navas et al.^(^3^)^ decidimos descrever o impacto da Pandemia de Covid-19 para a divulgação científica, no caso particular da revista CoDAS, a partir da comparação de indicadores da CoDAS pré e pós pandemia.

A utilização das métricas alternativas ou *altmetrics* (ALTernative article-level metrics) continua sendo considerada como estimativa do impacto promovido por determinado produto científico^(^5,6^)^. A partir dos artigos publicados em Acesso Aberto diferentes fontes geradoras de dados rastreiam o movimento do material científico no meio digital, quantificando o número de visualizações online, as leituras do texto na íntegra, número de downloads e menções em blogs ou mídias sociais^(^7^)^. Os *insights* fornecidos pelas mídias sociais permitem a compreensão do alcance e impacto de um artigo científico em dimensões diferentes em relação às métricas bibliométricas tradicionais^(^2^)^.

O uso das métricas alternativas possuem vantagens como a velocidade de disseminação e retorno quanto ao impacto do artigo científico e os elementos sociais promovidos pelo material (e.g. uso em políticas públicas, matérias em jornais para amplo público). Entretanto, é necessário ressaltar que ainda existem desafios no estudo das métricas alternativas, mas que não inviabilizam seu uso, como a falta de uma padronização e consistência entre as diferentes fontes que coletam, agregam e analisam os dados^(^8^)^. Ainda que, as métricas alternativas não substituam as métricas tradicionais, com certeza somam-se a estas para fornecer novas dimensões do impacto de determinado artigo científico na comunidade científica e social^(^2^)^.

Diante destes pressupostos, a Equipe de divulgação CoDAS continua com a divulgação de artigos publicados online e realiza o monitoramento em plataformas digitais, como um indicador importante do impacto da produção científica para a comunidade especializada e o público em geral.

As atividades da Revista CoDAS nas mídias sociais tiveram início em meados de 2019 e até o ano de 2020 tinham sido realizadas 69 publicações referentes a artigos científicos publicados nesta Revista nas redes sociais. Neste momento, as publicações referentes a artigos científicos totalizavam 394 nas redes sociais Instagram, Twitter e Facebook e 36 publicações no LinkedIn, rede social mais recente, criada no ano de 2023.

No Instagram, entre 2019 e 2020 a CoDAS apresentava 1.715 seguidores ativos com alcance médio de 1.900 contas e 2.600 impressões por publicação. Em 2023, o número de seguidores ativos aumentou para 5.060, o alcance médio de publicações para 4.505 e o número médio de impressões foi de 2.841, com aumento substancial de todos os indicadores.

Na conta do Twitter, também criada em 2019, havia 57 seguidores e número médio de 230 impressões por publicação, em 2020. E no Facebook, a página oficial da CoDAS tinha 1.231 seguidores ativos com alcance médio por publicação de 200 contas, até 2020. Em 2023, o número de seguidores ativos no Twitter foi de 118 e o número médio de impressões por publicação foi de 507. No Facebook o número atual de seguidores é de 2.200, com diminuição do número de alcance médio de publicação para 147. É necessário ressaltar que em âmbito nacional o uso de Facebook, em geral, diminuiu nos últimos anos, com seus usuários migrando para o Instagram.

A criação de uma conta oficial no LinkedIn foi pautada na importância desta rede social para relações de trabalho e acadêmicas. As atividades no LinkedIn iniciaram-se em Julho de 2023 e foram realizadas até o momento 38 postagens referentes a artigos científicos, com alcance médio de 1.193. São 432 seguidores ativos, com média de 60 visualizações do perfil por semana.

O Instagram permite a verificação do número de impressões (vezes que a publicação foi vista), o número de contas alcançadas, a taxa de engajamento e os dados demográficos. Nesta rede a publicação que promoveu o maior número de impressões no ano de 2022 foi o artigo de Mouffron et al.^(^9^)^, intitulado de “Efeitos imediatos da fotobiomodulação sobre a pressão máxima dos lábios”. A publicação que atingiu mais contas foi a de Nasciutti et al.^(^10^)^ com o artigo “Qualidade de vida do fonoaudiólogo brasileiro frente à pandemia da COVID-19”. Estes mesmos artigos foram os que receberam o maior número de curtidas no ano de 2022, seguido do “Disfagia por osteofitose cervical anterior: relato de caso” de Aires et al.^(^11^)^. No ano de 2023, a publicação que gerou o maior número de impressões foi “Mentira terapêutica: a visão do fonoaudiólogo brasileiro sobre uma estratégia de comunicação controversa no atendimento ao paciente com demência” de Lopes et al.^(^12^)^. O artigo de Santos et al.^(^13^)^ intitulado de “Efeito da frenotomia lingual na amamentação e na atividade elétrica dos músculos masseter e supra-hióideos” promoveu o maior número de contas alcançadas. O maior número de curtidas ocorreu na publicação do artigo “Efeitos do Método Canguru no desenvolvimento das habilidades orais e na aquisição da alimentação oral exclusiva em recém-nascidos pré-termo” de Ciochetto et al.^(^14^)^. No LinkedIn os *insights* fornecidos pela rede também se baseiam no número de contas alcançadas e impressões. A publicação que promoveu maiores números foi “Protocolo de avaliação para apraxia de fala adquirida” de Costa et al.^(^15^)^.

O Twitter fornece dados quanto ao engajamento por publicação, no ano de 2022 a Carta ao Editor de Dornelas et al.^(^16^)^ intitulada de “Tosse Crônica e Fonoaudiologia” promoveu os maiores números. No ano seguinte, a publicação com maior número de engajamento foi “Versão para Web do protocolo de avaliação miofuncional com escores: usabilidade e aprendizado” de Ataide et al.^(^17^)^. No Facebook, o artigo com maior número de engajamento de publicação em 2022 foi o mesmo que o Instagram, enquanto em 2023 o artigo de Ferrari et al.^(^18^)^ denominado de “Risco de disfonia e qualidade vocal em estudantes de artes cênicas”.

Considerando as diferentes áreas da fonoaudiologia, os anos de 2022 e 2023 e todas as redes sociais da revista, destacamos a publicação com maior número de compartilhamentos em cada área específica (Quadro 1).

**Quadro 1:** Publicações com maior número de compartilhamentos por área da Fonoaudiologia

**Área de Especialidade**	**Título do Artigo**	**Autoria**
Voz	Reduzindo o GAP entre a ciência e a clínica: lições da academia e da prática profissional - parte A: julgamento perceptivo-auditivo da qualidade vocal, análise acústica do sinal vocal e autoavaliação em voz.	Behlau et al.^(^19^)^
Audição e Equilíbrio	Síndrome da fragilidade e riscos para quedas em idosos da comunidade	Taguchi et al.^(^20^)^
Motricidade Orofacial	Efeitos imediatos da fotobiomodulação sobre a pressão máxima dos lábios.	Mouffron et al.^(^9^)^
Disfagia	Disfagia por osteofitose cervical anterior: relato de caso	Aires et al.^(^11^)^
Fala	Tradução e adaptação transcultural do Apraxia of Speech Rating Scale 3.5 para o português brasileiro.	Santos et al.^(^21^)^
Linguagem	Eficácia no uso de estratégia de gamificação na terapia fonológica	Silva et al.^(^22^)^
Fonoaudiologia Educacional	Pontos de corte, sensibilidade e especificidade para rastreamento da fluência leitora em crianças.	Cogo-Moreira et al.^(^23^)^
Saúde Coletiva	Apoio matricial para agentes comunitários sobre os marcos do desenvolvimento auditivo e de linguagem na primeira infância.	Silva e Silva^(^24^)^

A missão da SBFa é estimular, debater e divulgar a produção científica e profissional da área, bem como discutir questões relacionadas à formação em Fonoaudiologia e neste sentido a CoDAS tem sido um patrimônio valioso para a disseminação de evidências científicas para a prática profissional, para discussão de políticas públicas e para o fortalecimento da fonoaudiologia.
